# Prey capture strategies relate to prey position in the scale-eating cichlid *Perissodus microlepis*

**DOI:** 10.1242/bio.062699

**Published:** 2026-07-20

**Authors:** Kai Koike, Matasaburo Fukutomi, Yuichi Takeuchi

**Affiliations:** ^1^ Department of Marine Biology, Faculty of Fisheries Sciences, Hokkaido University, Hakodate, 041-8611, Japan; ^2^ Department of Biological Sciences, Faculty of Science, Hokkaido University, Sapporo, 060-0810, Japan

**Keywords:** Laterality, Feeding strategy, Predator–prey interaction, DeepLabCut, Scale eater, Cichlid

## Abstract

Predators have evolved sophisticated behavioral patterns that enable efficient prey capture. The scale-eating cichlid *Perissodus microlepis* exhibits pronounced morphological asymmetry in mouth-opening direction, corresponding to behavioral laterality in attack direction. Given that recognizing and tracking the flank of prey are crucial for successful predation, we hypothesized that the morphological asymmetry also influences their spatial positioning relative to prey during attack initiation. To test this idea, we simultaneously tracked predator and prey movements using DeepLabCut and analyzed the predatory sequence. Not only strike direction but also the lateralized approach initiation position was correlated with mouth asymmetry. Additionally, trajectory analyses revealed two distinct predation strategies based on the predator's relative position to the prey at the start of the approach: a rear approach, whereby predators move in from behind and strike at close range with strong body flexion, and a side approach, whereby predators initiate from lateral positions at shorter distances and rush forward. Although rear and side attacks differ in their kinematics and strike timing, both strategies achieve high predation success by applying strong forces to the prey's flank. Overall, this study advances our understanding of the predation strategies of scale-eating cichlids, providing insights into their evolutionary significance.

## Teaser

**Summary:** Mouth asymmetry influences both attack direction and approach positioning in scale-eating cichlids, which employ two distinct but equally successful predation strategies.

## INTRODUCTION

Capturing prey is fundamental to animal survival and has been a central focus in studies of the evolution of morphological and physiological traits (Caro, 2005; Higham et al., 2016). Predation typically involves a sequence of dynamic interactions – detection, evaluation, pursuit, and subjugation – resulting in either prey capture or escape (Curio, 2012; Endler, 1986). Although the strength of selective forces acting on predators and prey may differ, both exert strong influences on individual fitness. Consequently, predators have evolved sophisticated behavioral strategies to maximize prey capture efficiency. Foraging strategies range along a continuum from ambush to active pursuit. Predators that chase moving prey must process complex environmental cues while executing precise movements to ensure successful capture. For example, larval zebrafish visually detect prey and converge their eyes to form a small binocular strike zone in front of the head (Bianco et al., 2011; Patterson et al., 2013). Subsequently, they target their prey by closing the distance along the body axis and attack once the prey enters the strike zone (Borla et al., 2002; McElligott and O'Malley, 2005). Dragonflies employ an interception strategy in which they approach the prey from below, within the prey's blind spot, while stabilizing the prey's retinal image through predictive head rotations (Mischiati et al., 2015). In contrast, cuttlefish flexibly alternate between rapid tentacle ejection and full-body jump attacks depending on prey size and movement: small, fast-moving prey are typically captured by tentacle strikes, whereas prey like larger crabs elicit the jump strategy (Duval et al., 1984). Across taxa, vision serves as one of the most powerful and effective sensory modalities for prey capture, enabling predators to detect, pursue, and capture prey over a wide range of sizes and ecological contexts (Finger, 2000; Hairston et al., 1982; Kanwal and Finger, 1997).

The outcome of a predator's attack depends not only on the accuracy and velocity of the strike (Clark et al., 2012) but also on the evasive and escape behaviors of the prey (Clemente and Wilson, 2016; Combes et al., 2012; Moore and Biewener, 2015; Moore et al., 2017). When one participant – predator or prey – possesses superior speed, the other must compensate through enhanced locomotor performance (e.g. acceleration and maneuverability) or through behavioral strategies such as anticipatory positioning, stealth, or responsiveness (DeVries et al., 2012; Wilson et al., 2018). Thus, understanding the determinants of predator–prey encounter outcomes requires simultaneous analysis of the movement dynamics of both parties, yet such studies remain relatively rare.

Lake Tanganyika in East Africa harbors a highly endemic freshwater fauna and exhibits well-defined predator–prey relationships (Lowe-McConnell, 1987). More than 250 species of cichlids inhabit this lake, exhibiting extraordinary trophic diversification (Brichard, 1989; Greenwood et al., 1984; Iles and Fryer, 1972; Poll, 1956, 1986). These include aufwuchs feeders, planktivores, detritivores, shrimp eaters, benthic invertebrate feeders, piscivores, and scale eaters (Kovac et al., 2019; Muschick et al., 2012; Sturmbauer et al., 1992, 2010; Takeuchi et al., 2010). Among these trophic types, the scale-eating cichlids occupy a high trophic position, feeding on scales removed from the bodies of other fishes (Hori, 1987). The shallow-water species *Perissodus microlepis* is particularly well known and has been reported to attack 38 species of fish, primarily aufwuchs feeders such as *Tropheus* and *Petrochromis* (Nshombo et al., 1985). Therefore, *P. microlepis* plays a key ecological role as a predator influencing the fish community structure in Lake Tanganyika.

*P. microlepis* primarily recognizes and tracks its prey using visual cues (Takeuchi et al., 2012). Lefty individuals, whose mouths are twisted clockwise as viewed from above, preferentially attack the prey's left flank, whereas righty individuals, whose mouths are twisted counterclockwise, preferentially target the right flank. Because the scales targeted by *P. microlepis* are located on the sides of the prey, correctly recognizing the prey posture and body orientation during an attack is crucial for successful scale removal. Notably, this species exhibits lateralized predation: lefty individuals preferentially attack the prey's left flank, while righty ones target the right. This behavioral laterality has been consistently documented in both field (Hori, 1993; Lee et al., 2012) and laboratory conditions (Takeuchi et al., 2012). Kinematic analyses have shown that predation behavior consists of five sequential components: approaching the prey, moving stealthily toward the prey's flank, stopping for a split moment in an S-shaped posture, flexing the body quickly, and twisting. Attacks from the dominant side produce larger and faster flexions, resulting in higher predation success (Takeuchi et al., 2012). This attack-side preference strongly suggests that predation behavior is fine-tuned according to the prey's position and movement. However, previous studies have focused exclusively on predator behavior (Takeuchi et al., 2022, 2025, 2012; Takeuchi and Oda, 2017), analyzing its relationship with predation dynamics and laterality. Similarly, in most typical studies involving freely swimming fish, a single behavioral component has been quantified (Juanes and Conover, 1994; Webb, 1986). Simultaneous quantification of the locomotor performance in both predator and prey remains rare, with the exception of guppy–pike cichlid interactions (Milton and Bergmann, 2023; Walker et al., 2005). Recent advances in deep-learning-based analytical tools, such as DeepLabCut (Mathis et al., 2018; Nath et al., 2019), have enabled the simultaneous quantification and analysis of the movements of multiple individuals.

In this study, we aimed to elucidate how the scale-eating cichlid modulates its predatory strategy in response to prey behavior by tracking the positions and kinematics of both predator and prey. First, we hypothesized that mouth asymmetry would influence not only strike direction but also the spatial positioning of predators during approach initiation. Specifically, we predicted that lefty and righty individuals would preferentially initiate approaches from positions corresponding to their dominant attack side; for example, lefty individuals were expected to approach from the left rear quadrant relative to the prey. To test the idea, we reanalyzed high-speed video recordings from a previous study (Takeuchi et al., 2012) using DeepLabCut. An examination of the predator's trajectory revealed two distinct predation strategies based on its relative position to the prey. Analyses of predator trajectories identified two distinct predation strategies based on the predator's relative position to the prey: a rear approach and a side approach. We then hypothesized that these different approach angles would involve distinct combinations of translational and rotational movements during the strike phase. Finally, we compared predator–prey timing relationships and predation success across these strategies to clarify their functional differences.

## RESULTS

### Relationship between approach position and asymmetrical mouth morphology

**Fig. 1.**
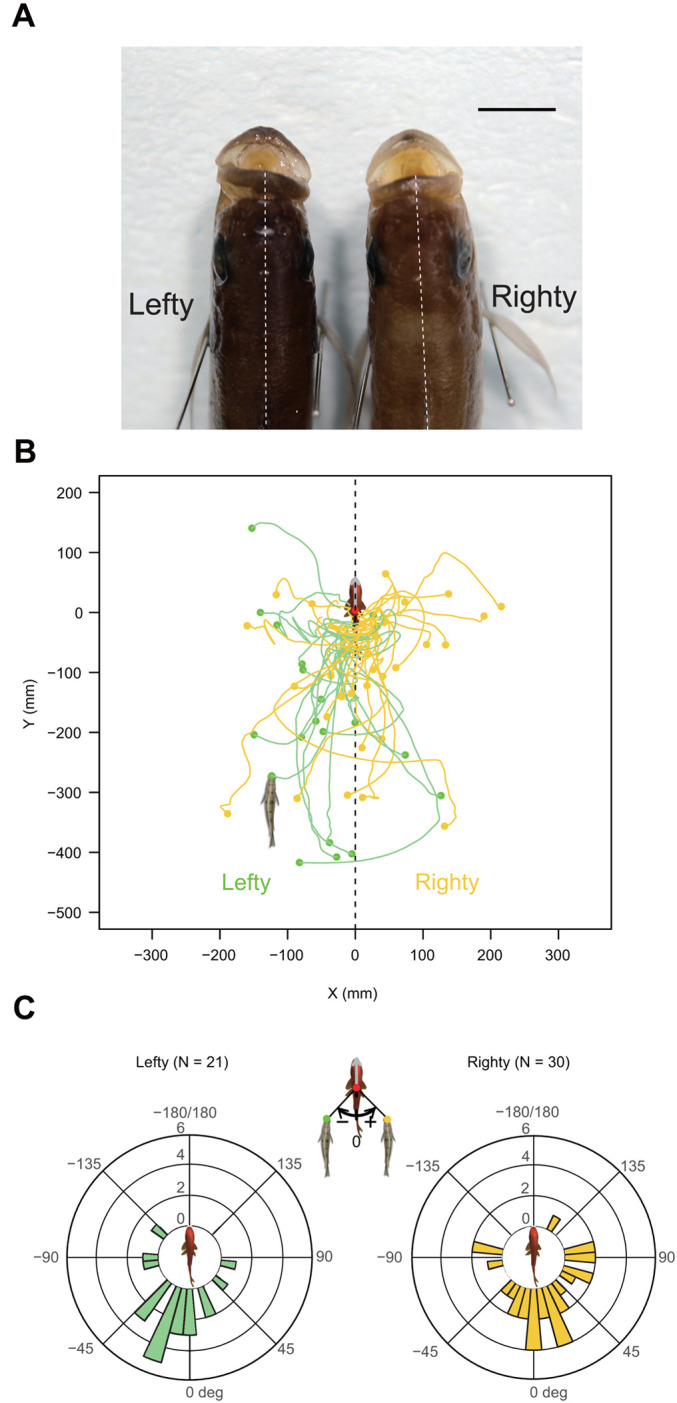
**Approach position related to asymmetrical mouth morphology.** (A) Morphological mouth asymmetry in the scale-eating cichlid *P. microlepis*. Dorsal views of representative lefty and righty individuals. Dotted lines indicate the body midline. The mouth opening deviates to the right in lefty fish and to the left in righty fish. Scale bar: 1 cm. (B) Approach trajectories plotted in a prey-aligned coordinate space, colored according to morphological asymmetry (lefty: green, *N*=30; righty: yellow, *N*=21). Dots represent relative positions at approach start, and solid lines indicate trajectories from approach start to strike finish. The vertical dashed line represents the *y*-axis aligned to the prey's body axis. (C) Distribution of approach angles. Polar histograms of relative angles at approach start for each mouth asymmetry type. Righties predominantly started approaching from the prey's right side, and lefties from the left side. Bin width=10°.

We divided the scale-eating cichlid predatory sequence into two phases – an approach phase and a strike phase – based on head speed and the angular velocity of body-axis bending. We found that not only the strike direction but also the lateral bias of the approach initiation position was correlated with asymmetric mouth morphology (*N*=30 lefty individuals; *N*=21 righty individuals; Fig. 1A). Analysis of the relative angle between prey and predator revealed that lefty individuals tended to initiate their approach more frequently from the prey's left side (19/30), whereas righty individuals more frequently initiated their approach from the right side (17/21). Consistent with this trend, mouth morphology was significantly associated with initiation side (chi-squared test: χ^2^=8.08, *P*=0.005; Fig. 1B). This showed that both mouth asymmetry and the spatial relationship to the prey are important in decision-making at approach initiation.

### Identification of two predation strategies based on approach trajectories

**Fig. 2.**
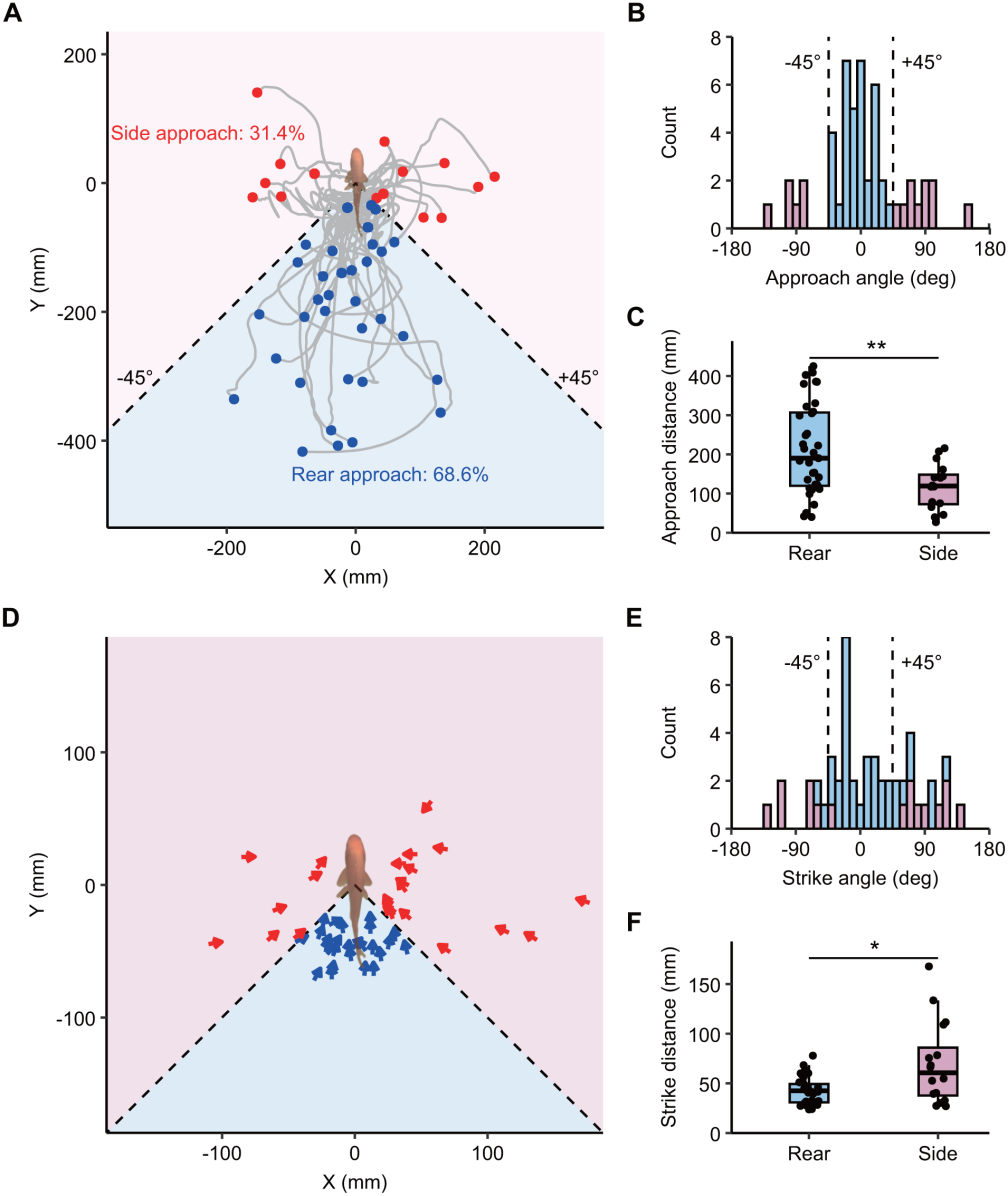
**Two predation strategies: rear and side approaches.** (A) Definition of predation strategies based on approach angle. The trajectories were categorized into a rear approach (*N*=35) and side approach (*N*=16) using a threshold of ±45° from the prey midline (0°=directly behind the prey; positive values indicate positions on the prey's right side). (B) Histogram of approach angle (bin width=10°). (C) Boxplot of approach distance, defined as distance between the prey's trunk center and the snout point of *P. microlepis*. Rear approaches were initiated from significantly greater distances than side approaches. (D) Relative predator positions at strike start. Arrows indicate the orientation of the predator, represented by a normalized vector from the trunk center to the snout (blue arrows: rear approach, red arrows: side approach). (E) Histogram of strike angle (bin width=10°). (F) Comparison of strike distances. Rear approaches had significantly shorter strike distances than side approaches. All *P*-values were obtained from Wilcoxon signed-rank tests. Asterisks denote statistical significance (*P<0.05, **P<0.01).

In addition to laterality, analysis of approach trajectories revealed two predation strategies: approaching from behind and approaching from the side (Fig. 2A). Based on the distribution of approach angles (Fig. 2B), we classified the trajectories into rear approaches (*N*=35) and side approaches (*N*=16) using a threshold of ±45°. The rear approach was initiated from significantly greater distances than the side approach (rear approach: 213±116 mm, side approach: 118±59 mm; Wilcoxon rank-sum test, *Z*=2.68, *P*=0.007; Fig. 2C). Strikes during rear approaches occurred within the relative angle of ±45°, whereas strikes during side approaches occurred outside this range. Accordingly, the distribution of strike positions closely resembled that of the approach angles. Strike distance also differed significantly between the two strategies, with rear approaches resulting in shorter strike distances than side approaches (rear: 42.9±13.5 mm, side: 69.7±41.6 mm; Wilcoxon rank-sum test, *Z*=−2.13, *P*=0.033; Fig. 2F). In addition, the variance in strike distance was significantly smaller for the rear approach than for the side approach (Levene's test, *F*=17.5, *P*<0.001). Thus, rear approaches involved strikes initiated at shorter distances from the prey, whereas side approaches involved strikes initiated over a wider range of distances.

### Kinematic differences between predation strategies

**Fig. 3.**
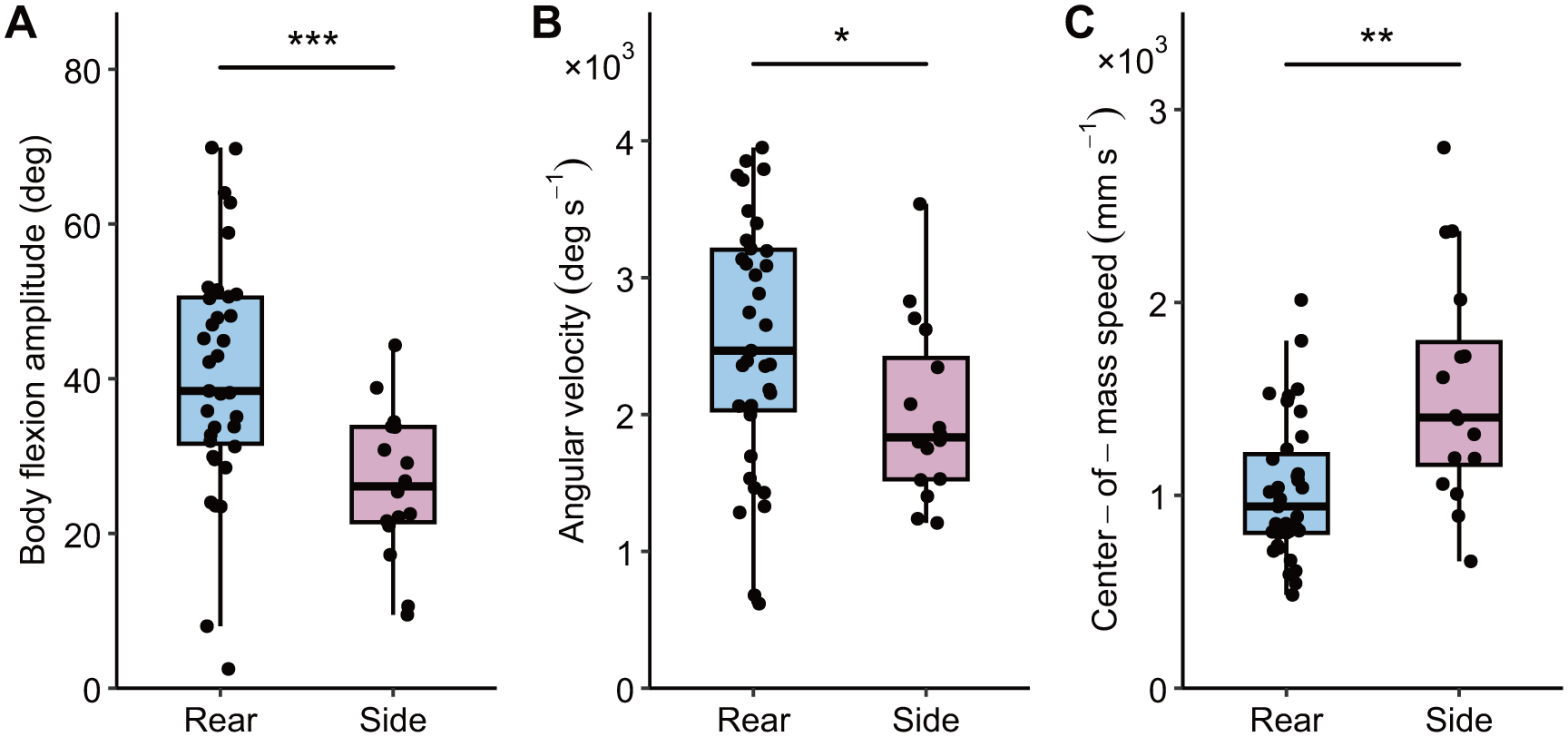
**Kinematic differences between rear and side approaches.** (A) Maximum body flexion amplitude during the strike phase. Body flexion amplitude was significantly greater in rear approaches (*N*=35) than in side approaches (*N*=16). (B) Maximum angular velocity during the strike phase. Angular velocity was significantly higher in rear approaches than in side approaches. (C) Maximum center-of-mass speed during the strike phase. Center-of-mass speed was significantly higher in side approaches than in rear approaches. For each boxplot, the bottom and top of the box indicate the 25th and 75th percentiles, respectively; the thick black line indicates the median; and the whiskers extend to the most extreme data points that are not outliers. Outliers are defined as values more than 1.5 times the interquartile range beyond the lower or upper quartile. All *P*-values were obtained from Wilcoxon signed-rank tests. Asterisks denote statistical significance (*P<0.05, **P<0.01, ***P<0.001).

We next compared predator kinematics during the strike phase between rear- and side-approach strategies. In particular, we compared body flexion amplitude, maximum angular bending velocity, and maximum center-of-mass speed, all of which were quantified in the original coordinate space (see also Materials and Methods). Body flexion amplitude was significantly greater in the rear approach than in the side approach (rear approach: 40.5±15.3°, side approach: 26.4±9.6°; Wilcoxon rank-sum test, *Z*=3.37, *P*<0.001; Fig. 3A). Similarly, maximum angular velocity was higher in the rear approach (rear approach: 2530±908° s^−1^, side approach: 2010±645° s^−1^; Wilcoxon rank-sum test, *Z*=2.21, *P*=0.027; Fig. 3B). In contrast, maximum center-of-mass speed was significantly higher in the side approach (1550±594 mm s^−1^) than in the rear approach (1030±365 mm s^−1^; Wilcoxon rank-sum test, *Z*=−3.19, *P*=0.001; Fig. 3C). These results indicate that the rear approach is characterized primarily by rotational motion generated through strong body flexion, whereas the side approach relies more on translational motion.

### Relative timing between predator strike and prey escape

**Fig. 4.**
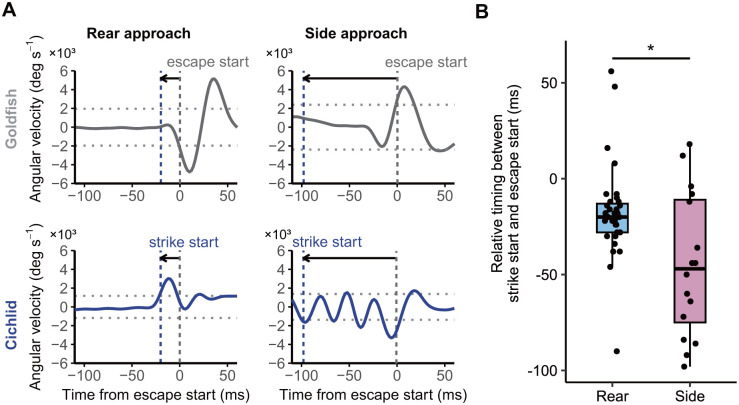
**Relative timing of predator strikes and prey escapes.** (A) Example of predator–prey interactions in each predation strategy. Angular velocity of the cichlid predator (blue line) and that of the goldfish prey (gray line) are shown with dotted threshold lines (±2 s.d.). Gray and blue vertical lines indicate the onset of the predator's strike and the prey's escape response, respectively. Arrows denote relative timing (strike start−escape start). (B) Comparison of relative timing between strategies. Delays were significantly shorter in rear approaches than in side approaches. The *P*-value was obtained from a Wilcoxon signed-rank test. Asterisk indicates statistical significance (*P<0.05).

To investigate how the two strategies relate to prey behavior, we compared the timing of strike start relative to the onset of the prey escape response. Relative timing values were predominantly negative in both strategies, indicating that predators typically initiated strikes before prey began to escape regardless of the strategy. Side-approach strikes began significantly earlier relative to prey escape than those in rear approaches (rear approach: −17.7±24.1 ms, side approach: −45.2±37.4 ms; Wilcoxon rank-sum test, *Z*=2.3, *P*=0.021; Fig. 4B). Furthermore, the variance in relative timing was significantly smaller for the rear approach than for the side approach (Levene's test, *F*=7.57, *P*=0.008). These findings indicate that predator–prey interactions during rear approaches are more temporally stereotyped, whereas side approaches show greater variability in the timing between predator strike and prey escape.

### Predation success and its determinants

**Fig. 5.**
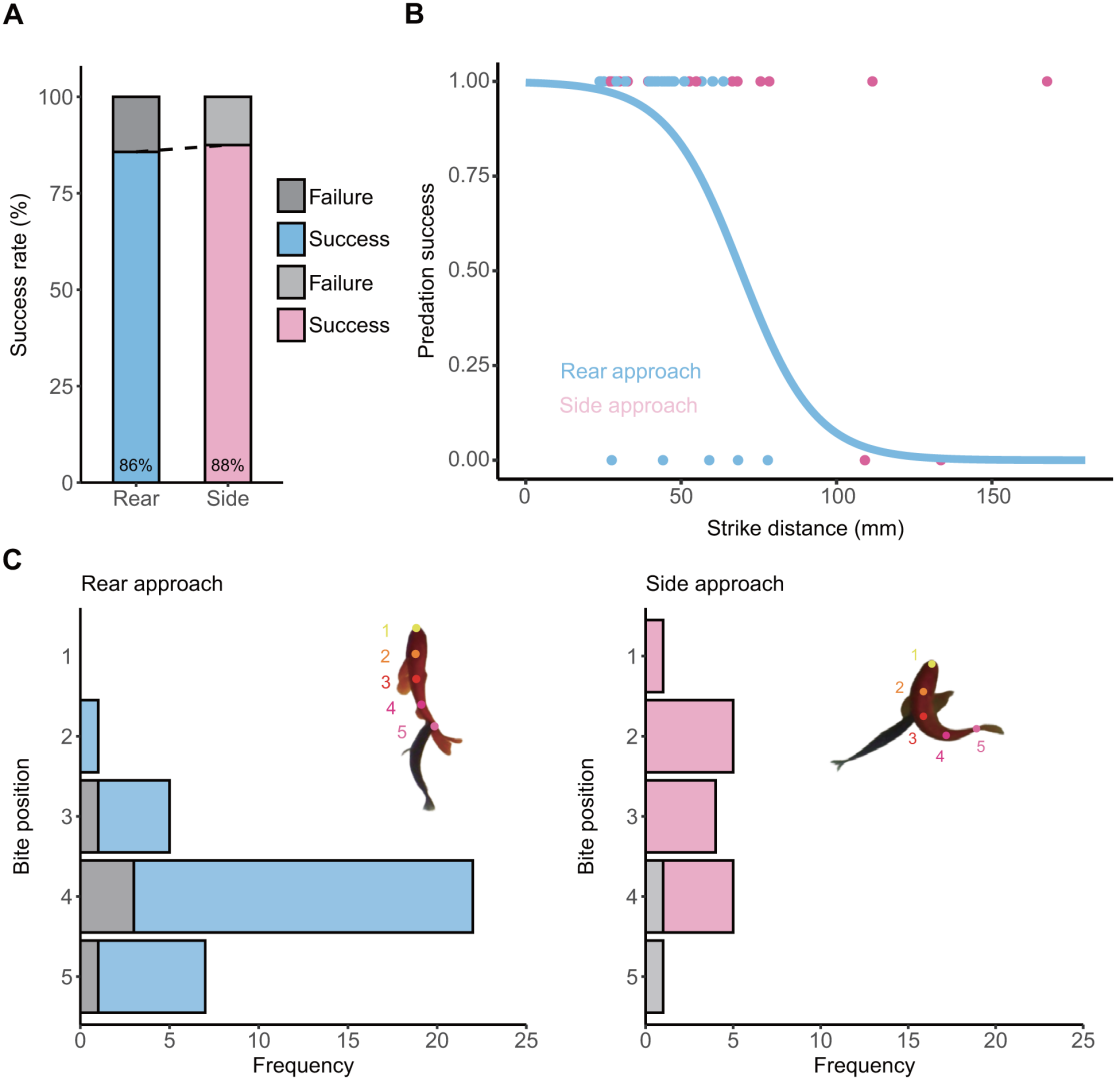
**Factors affecting predation success.** (A) Predation success rates of rear and side approaches. (B) Relationship of predation success and strike distance. The logistic regression curve displays the predicted probabilities of predation success as a function of strike distance. The predictions were obtained from a binomial GLM including strike distance, predation strategy (rear versus side approach), and their interactions as explanatory variables. Each dot represents an individual trial (1=success, 0=failure). (C) Distribution of bite positions along the prey body axis by predation outcome. Insets show examples of *P. microlepis* biting at position 5 (left) and position 3 (right).

Finally, we evaluated whether differences in the predation strategies influenced predation outcome. Predation success rate did not differ between the strategies (rear: 86%, *N*=35; side: 88%, *N*=16; Fig. 5A). To identify factors influencing predation outcome, we fitted a generalized linear model (GLM) (Table 1). This revealed that attack distance had a significant negative effect on predation outcome (*P*=0.041; Fig. 5B), indicating that shorter strike distances increased the probability of predation success. However, neither the strategy (*P*=0.085) nor the interaction between strike distance and strategy (*P*=0.276) had a significant effect. Therefore, shorter strike distances improved capture success, regardless of strategy type.

**Table 1.** ANOVA results for a GLM testing the effects of strike distance, predation strategy, and their interaction on predation outcome

Model term	df	Deviance	Residual df	Residual deviance	*P*-value
Strike distance	1	4.195	49	36.599	0.041
Predation strategy	1	2.975	48	33.624	0.085
Distance×strategy	1	1.187	47	32.438	0.276

In successful trials, bite position also differed between strategies; the rear approach primarily targeted the caudal region of the prey (position 5), whereas the side approach more frequently targeted the anterior–central body region (positions 2 and 3) (Fig. 5C). In failed trials, bite position was concentrated on the caudal region for both strategies.

## DISCUSSION

Our analyses supported the hypothesis that asymmetric mouth morphology influences not only attack direction but also spatial positioning during approach initiation. In addition, trajectory analyses of predator and prey identified two distinct predation strategies that differed in locomotor patterns and predator–prey timing relationships. The rear approach was initiated from behind the prey at a relatively long distance, and the attack was executed at close range through rapid body flexion. This strategy exhibited stereotypical movement patterns relative to prey motion and accounted for 69% of all attacks. In contrast, the side approach involved predators moving in from the lateral side of the prey and removing scales via a forward rushing movement, often initiating the strike before the onset of the prey's escape response. Side approaches comprised 31% of all attacks. Despite their distinct kinematic characteristics, both strategies were highly effective, with no significant difference in predation success (rear: 86%, side: 88%). In other predatory fish species, different predation strategies have often been associated with prey distance when the attack is initiated (Patterson et al., 2013; Webb and Skadsen, 1980). In the scale-eating cichlid, however, strategies were clearly determined by the angle of approach. This likely reflects the unique feeding ecology of scale-eating fish, in which the target is the prey's flank; therefore, accurately positioning relative to the prey's orientation is critical for successful scale removal.

Behavioral laterality during predation is widespread among fishes including cichlids, black bass, and anglerfishes (Hata et al., 2011; Nakajima et al., 2007; Takeuchi and Hori, 2008; Yasugi and Hori, 2012, 2016) and is thought to enhance capture success by enabling specialization in unilateral attacks (Hata et al., 2011; Kurvers et al., 2017; Yasugi and Hori, 2012). Such laterality in feeding behavior appears particularly pronounced in predators targeting prey with strong escape abilities (Takeuchi et al., 2019). The laterality has been quantified as a binary variable, representing attack direction as either right or left. However, it is important to note that the laterality is also related to the timing of prey detection and escape responses, thereby influencing predation success (Takeuchi et al., 2025; Yasugi and Hori, 2012). We found that predatory laterality is associated with the spatiotemporal relationship between predator and prey. In addition to laterality, time-series analysis of predatory behavior showed that the two different strategies are also related to prey position. Applying such analyses to other predatory species may reveal new aspects of predator–prey interactions.

Rear and side approaches differed not only in initial position but also in their movement kinematics. During the rear approach, the predator approached stealthily from behind and adjusted toward the dominant side immediately before striking. At the moment of attack, the body axes of predator and prey were nearly parallel, and scales were removed primarily through the predator's rapid body flexion. In contrast, during the side approach, the body axes of predator and prey were nearly perpendicular, and the strike was dominated by the predator's translational motion. Despite these distinct movement patterns, both strategies enable effective scale removal by applying strong forces to the prey's flank. In both strategies, predation success increases when the predator is able to attack from a closer range. The rear approach achieves high success likely because the stealthy approach from behind reduces the chance of being detected by the prey and enables close-range attacks powered by body flexion. In addition, the movement pattern relative to the prey in the rear approach appears to be more stereotypical than that of the side approach, suggesting a sophisticated strategy. In the side approach, the success rate may be enhanced because this strategy more frequently targets the anterior–central region of the prey's body (Fig. 5C), where the prey cannot move its center of mass as easily or as rapidly compared to the head or tail during a fast-start escape response (Webb and Skadsen, 1980). Although approaching from the side increases the risk of detection, initiating the strike before the onset of the escape response and approaching at high speed likely compensate for this disadvantage. Thus, the rear and side approaches likely offer different tactical advantages.

Feeding behaviors are typically triggered by specific sensory cues, such as visual stimuli. For example, prey-catching behavior in toads is elicited by visual stimuli, with elongated objects moving along their long axis being recognized as worm-like prey (Ewert, 1987; Ewert et al., 2001). The scale-eating cichlids rely heavily on visual cues during predation (Takeuchi et al., 2012). We found that the position at which the approach was initiated corresponds to the mouth-opening direction. The eye on the mouth-opening side is known to be larger and more developed than the contralateral eye (Raffini et al., 2018), and this larger eye functions as the dominant eye during predation and escape behaviors (Takeuchi et al., 2025). These findings indicate that scale-eating cichlids initiate their approach when prey are detected within the visual field corresponding to the mouth-opening side, highlighting the importance of accurate visual assessment of prey orientation in triggering the predatory sequence. Furthermore, the visual image of the prey that triggers predatory behavior likely varies with predation strategy, suggesting that both the direction and type of approach are determined at the onset of the predatory sequence.

The neural mechanisms that control predation behavior are considered to be located in the visuomotor pathway. In zebrafish (*Danio rerio*) larvae, visually guided hunting depends on optic tectum–pretectal circuitry: neurons in pretectal arborization field 7 initiate hunting behavior, whereas the nucleus isthmi maintains pursuit (Antinucci et al., 2019; Henriques et al., 2019; Zhu and Goodhill, 2023). These findings suggest that such neural mechanisms may also be involved in determining behavioral lateralization and in selecting approach strategies in scale-eating fish. With the recent establishment of a chromosome-level reference genome for *P. microlepis* (Tian et al., 2025), this species provides a promising model for investigating the neural and genetic basis of prey recognition and decision-making during predation. Its pronounced morphological asymmetries and behavioral laterality make it particularly suitable for studying the evolution of lateralized behavior.

The scale-eating cichlids exhibit substantial individual variation in both mouth morphology and lateralized behavior (Marubayashi et al., 2025; Takeuchi et al., 2016), suggesting that predation strategies may also vary among individuals. Because the number of predation events per individual was limited in this study, we could not determine whether the observed strategies represent fixed individual traits or flexible behavioral options. Future studies should increase the number of trials per individual to clarify the degree of strategy flexibility and the mechanisms underlying potential switching between strategies. The rear and side approaches identified here differed in strike timing, locomotor dynamics, and bite location, suggesting that prey may experience them as distinct forms of attack. Such behavioral diversity could increase attack unpredictability and reduce the prey's ability to anticipate effective escape responses. Further, the ratio of lefty and righty individuals in *P. microlepis* populations is maintained by negative frequency-dependent selection (Hori, 1993). Variation in predatory behavior may contribute to maintaining this left–right dimorphism. Investigating the diversity of predation strategies within a species therefore has the potential to provide important ecological and evolutionary insights into complex predator–prey interactions.

## MATERIALS AND METHODS

### Data collection

**Fig. 6.**
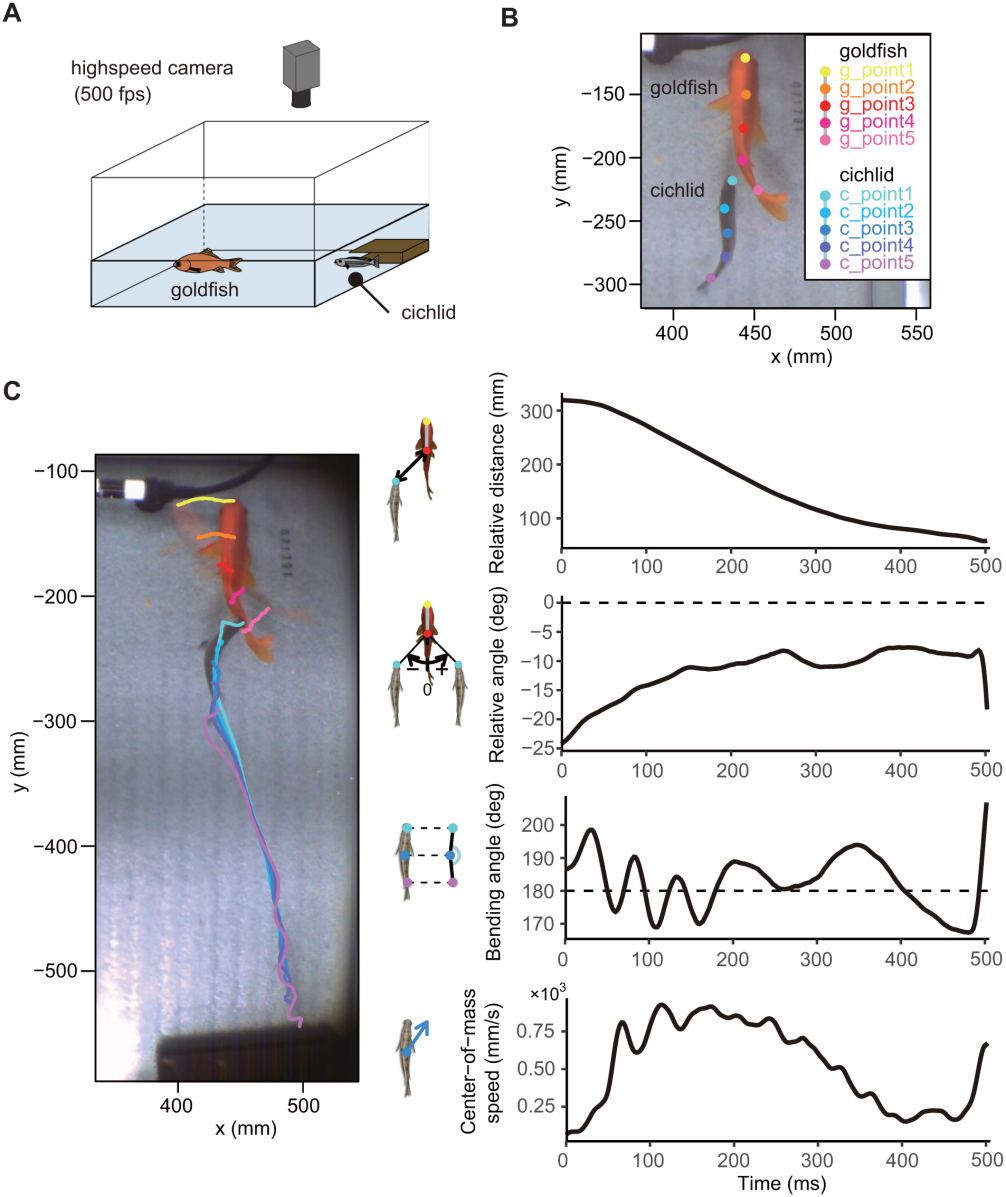
**Quantifications of predator–prey interactions.** (A) Schematic diagram of the experimental setup. The scale-eating cichlid *P. microlepis* was recorded from above using a high-speed camera while removing scales from a goldfish within the experimental arena. (B) Annotated body landmarks. Five equidistant points along the body axis of both *P. microlepis* (predator) and the goldfish (prey) were labeled using DeepLabCut (Mathis et al., 2018; Nath et al., 2019). (C) Example trajectories and kinematic variables. Left: Representative trajectories of *P. microlepis* and goldfish during a predation sequence. The *P. microlepis* individual is attacking the goldfish from the left side while the prey initiates an escape response. Right: Kinematic variables extracted from the coordinate data. The relative distance and angle from the center of the prey's body axis to the predator's snout, the body bending angle (defined by the snout, trunk center, and caudal peduncle), and center-of-mass speed.

We reanalyzed previously recorded high-speed video data of predation behavior of a scale-eating cichlid (*P. microlepis* Boulenger, 1898) on goldfish scales (*Carassius auratus* Linnaeus, 1758) (Takeuchi et al., 2012). Animal care procedures were approved by the Institutional Animal Care Committee of Nagoya University (permit number #0014) (Takeuchi et al., 2012). A total of 51 trials obtained from 16 cichlids were selected in which the movements of both *P. microlepis* and its prey (the goldfish) were clearly visible. Mouth asymmetry data were taken from previously published measurements (Takeuchi et al., 2012). Predation experiments were conducted in an acrylic tank (90×45×15 cm; length×width×depth) using one *P. microlepis* individual (standard length: 73±6 mm, mean±s.d.) and one goldfish (standard length: 100±11 mm, mean±s.d.) per trial (Fig. 6A). A brown plastic shelter was placed in one corner of the tank. The tank was illuminated with three HVC-SL video lights (Photron, Tokyo, Japan), and the fish behavior was recorded from above using a high-speed camera (FASTCAM 1024PCI, Photron, Tokyo, Japan) at 500 frames s^−1^ and a spatial resolution of 1024×1024 pixels.

### Capture pose estimation

To track the movements of *P. microlepis* and the goldfish, we used DeepLabCut (Mathis et al., 2018; Nath et al., 2019). For each fish, we labeled five key points equally spaced along the body: one at the snout (point 1), three evenly spaced points along the trunk (points 2–4), and one at the caudal peduncle (point 5) (Fig. 6B). Throughout this study, labeled points on the scale-eating cichlid and the goldfish are denoted as c_point1–c_point5 and g_point1–g_point5, respectively. Points with likelihoods estimated by DeepLabCut below 0.9 were manually corrected using Dipp Motion V (Ver. 1.1.25; Ditect, Tokyo, Japan). The final coordinate data were smoothed using a moving-average filter with a window size of nine frames to reduce high-frequency noise.

### Movement analysis

From the smoothed coordinate data, head speed, center-of-mass speed, body bending angle, and angular velocity were calculated. Head speed and center-of-mass speed were calculated from the frame-to-frame displacement of the snout (point 1) and the trunk center (point 3), respectively. The body bending angle was defined as the angle at the trunk center formed by the head and the caudal peduncle (point 5). Angular velocity was obtained as the time derivative of this angle (Fig. 6C). Head speed, center-of-mass speed, and angular velocity data were further filtered using a low-pass Butterworth filter with a cutoff frequency of 100 Hz.

### Coordinate transformation for relative predator–prey position

**Fig. 7.**
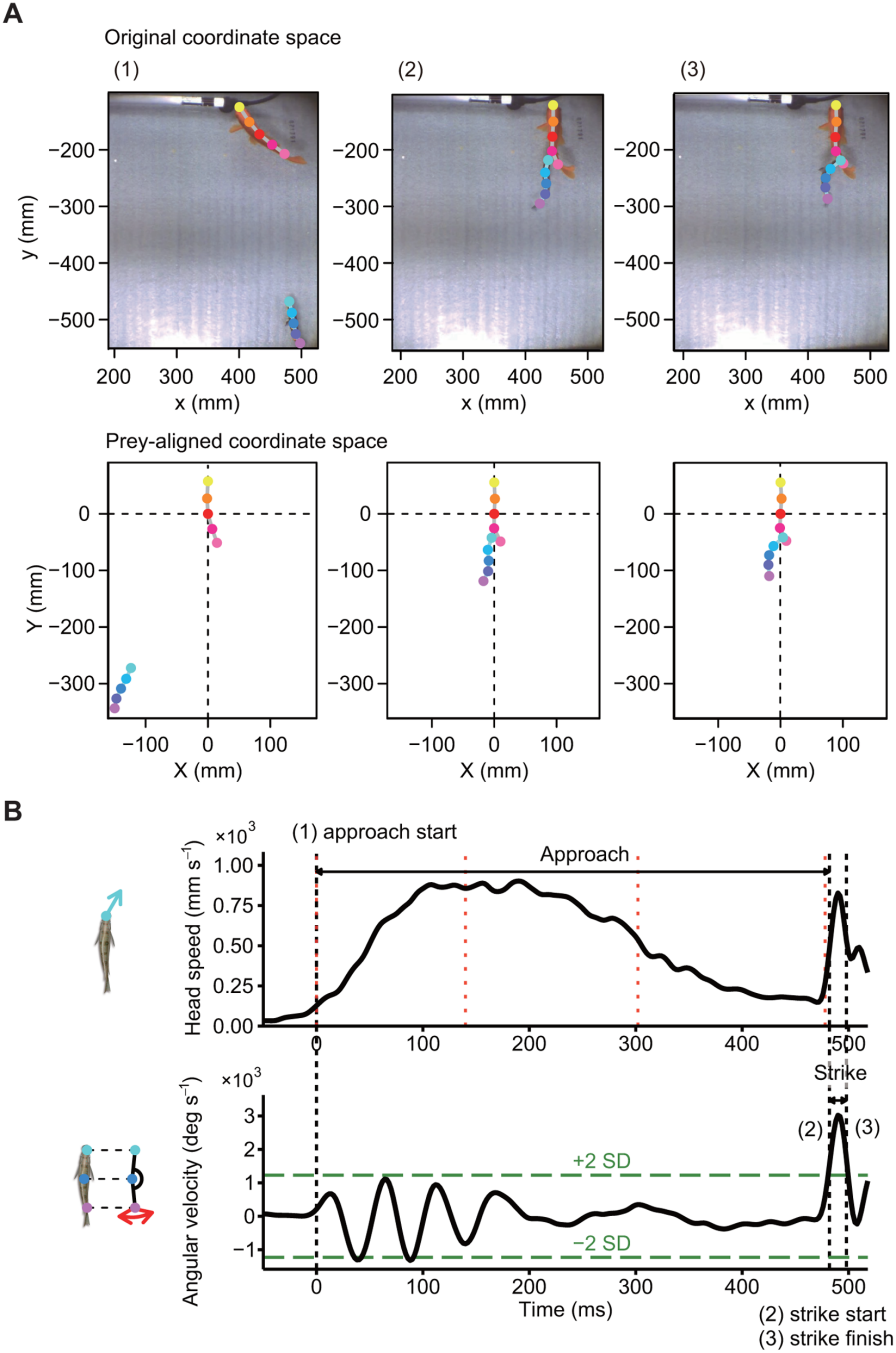
**Identification of behavioral phase transitions.** (A) Prey-aligned coordinate space. Top: Representative video frames of a predation sequence at three time points: (1) approach start, (2) strike start, and (3) strike finish. Bottom: Corresponding body positions plotted in a prey-aligned coordinate space at each time point. (B) Kinematic time series. Time course of head speed and angular bending velocity calculated in the original coordinate space were shown. Vertical black dashed lines mark the three behavioral transitions: (1) approach start, (2) strike start, and (3) strike finish. Approach start is defined as the change point in head speed detected using the R package strucchange (Zeileis et al., 2002, 2003). Red dotted lines indicate the detected change points. Strike start and strike finish are defined as the first and last frames exceeding the 2 s.d. threshold of angular velocity within the 100 ms preceding the moment of closest relative distance. Based on these three points, approach and strike phases were defined (indicated by black double-headed arrows). For details, see Materials and Methods.

To quantify the predator's position relative to the prey, we defined a prey-aligned coordinate system independently for each frame, with its origin at the prey's trunk center (g_point3) and its positive y-axis aligned with the trunk center–snout (g_point1) vector (Fig. 7A).

The rotation angle *φ*(*t*)was calculated as:
(1)

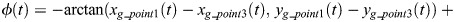
where 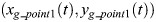 and 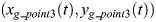 denote the coordinates of the goldfish head (g_point1) and trunk center (g_point3), respectively, at frame *t*.

Transformed coordinates (*X*_rotation_(*t*),  *Y*_rotation_(*t*)) of any tracked point (*X*(*t*), *Y*(*t*)) were obtained using the following rotation matrix:
(2)

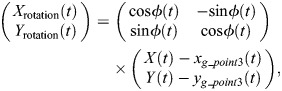
where (*X*(*t*), *Y*(*t*)) represents the coordinates of any labeled point on either the scale-eating cichlid or the prey goldfish.

These transformed coordinates were used only to quantify the instantaneous position of the predator relative to the prey and for the classification of predation strategies. Movement variables, including center-of-mass speed, body flexion amplitude, and angular velocity, were calculated in the original coordinate space.

### Behavioral phase classification

Each predation sequence was divided into two phases – approach phase and strike phase – based on three time points: (1) approach start, (2) strike start, and (3) strike finish (Fig. 7B). The approach start was determined using a linear model-based change point detection algorithm implemented in the R package strucchange (Zeileis et al., 2002, 2003). It was difficult to determine a change point using a fixed threshold, as the speed profiles exhibited substantial inter-individual variability. The strucchange algorithm yielded multiple segments. If the absolute slope of the initial segment exceeded the acceleration threshold (1500 mm s^−2^), the approach start was defined as the first time point within that segment at which the change in speed exceeded the threshold (17 out of 51 trials). If the absolute slope of the initial segment did not exceed the threshold, the end of the initial segment was used (34 out of 51 trials).

The strike phase was defined based on the angular velocity and relative distance. First, the frame corresponding to the minimum relative distance (i.e. closest approach) between predator and prey was determined. The standard deviation (s.d.) of angular velocity was then calculated from preceding frames during the pre-strike period. Within the 100 ms preceding the closest approach point, strike start and strike finish were defined as the first and last frames, respectively, in which angular velocity exceeded 2 s.d. In two trials where this threshold was not exceeded, a relaxed threshold of 1 s.d. was used. Similarly, prey escape start was identified as the first frame within ±100 ms of the closest approach where the prey's angular velocity exceeded 2 s.d. The temporal difference between strike start and escape start was defined as the relative delay (relative delay=strike start−escape start). Negative values indicate that the predator initiated the strike before the onset of the prey's escape response, whereas positive values indicate that the prey initiated escape before the predator's strike. During the strike phase, the maximum angular velocity, maximum center-of-mass speed, and maximum body flexion amplitude (defined as the maximum deviation angle from a fully extended tail posture) were extracted. The bite location was defined as the prey landmark (among the five labeled points) that was closest to the predator's head during the strike. Predation strategies were categorized into two types – rear approach and side approach – based on the approach angle, using ±45° from the prey's midline based on the landmarks as the boundary. Trial outcomes were defined following Takeuchi et al. (2012): a trial was considered successful if the predator's mouth contacted the prey's body and unsuccessful otherwise. The predation success rate for the rear approach and side approach was calculated as the proportion of successful trials within each strategy category.

From the transformed coordinates, the relative distance and relative angle of snout position to the prey were calculated, with 0° representing a position directly behind the prey and positive values indicating the right side (Fig. 6C). The relative position at the onset of each approach and strike was quantified in polar coordinates, yielding approach distance and angle, and strike distance and angle, respectively.

### Statistical analysis

Differences in approach distance, strike distance, kinematic parameters (maximum angular velocity, maximum body flexion amplitude, maximum center-of-mass speed), and relative delay time between strategies were tested using the Wilcoxon rank-sum test. Differences in the variance of strike distance and relative delay time were evaluated using Levene's test. To evaluate the effects of strike distance and predation strategy on trial outcome, a GLM (binomial distribution) was fitted. The dependent variable was trial outcome (success=1, failure=0), and the independent variables included strike distance, predation strategy (rear versus side approach), and their interaction. Model significance was evaluated using analysis of variance (ANOVA) with a significance level of α=0.05.

## Supplementary Material


