# Supplementary material for: Prey capture strategies relate to prey position in the scale-eating cichlid Perissodus microlepis
Source: Biol Open. 2026 Jul 20;15(7):bio062699. doi: 10.1242/bio.062699 (PMC13446557; doi:10.1242/bio.062699)
Supplement: Supplementary file 1 [file biolopen-15-062699_review_history.pdf]

Prey capture strategies relate to prey position in the scale-eating cichlid Perissodus microlepis

Kai Koike, Matasaburo Fukutomi and Yuichi Takeuchi
10.1242/bio.062699

Editor: Valentina di Santo

Review timeline

Original submission:	15 May 2026
Editorial decision:	22 May 2026
First revision received:	5 June 2026
Accepted:	15 June 2026

Original submission

First decision letter

MS ID#: bio.062699

MS Title: Prey capture strategies relate to prey position in the scale-eating cichlid Perissodus microlepis

Authors: Kai Koike; Matasaburo Fukutomi; Yuichi Takeuchi

I have now reached a decision on the above manuscript.

The reviewer reports are shown at the bottom of this email.

As you will see, the reviewers raised a number of substantial criticisms that prevent me from accepting the paper at this stage.

They suggest, however, that a revised version might prove acceptable, if you can address their concerns. If you think that you can deal satisfactorily with the criticisms on revision, I would be pleased to see a revised manuscript. We would then return it to the reviewers.

At this stage, we also ask you to ensure your manuscript complies with our formatting guidelines. Provided you are able to fully address the referees' comments, we are positive about publication of your paper (we accept over 95% of revision submissions) and therefore hope you won't mind any extra work involved in reformatting your manuscript at this point.

Please upload both a 'clean' version of your Word file, along with a highlighted version clearly showing where you have made changes in the revised manuscript. Please avoid using 'Track changes' in Word files as these are lost in PDF conversion.

I should be grateful if you would also provide a point-by-point response detailing how you have dealt with the points raised by the reviewers in the 'Response to Reviewers' box. Please attend to all of the reviewers' comments. If you do not agree with any of their criticisms or suggestions please explain clearly why this is so.

© 2026. Published by The Company of Biologists under the terms of the Creative Commons Attribution License (https://creativecommons.org/licenses/by/4.0/).

Reviewer 1

Comments for the author

This is a clear and comprehensive manuscript detailing a well-designed experiment. The methods are clear and enable reproducibility, and the conclusions draw directly from the results. I only have a few suggestions for improvements to the manuscript. Below I have structured my comments according to the BiO rubric:

Experimental quality

The experimental design is high quality, and well explained in the text. Appropriate statistical tests are chosen.

The figures are very clear, easy to interpret, and link explicitly to the text.

I think it would be worth briefly mentioning that the ethical considerations and permits were in line for the original data collection. After the sentence "We reanalyzed previously recorded high-speed video data of predation behavior of a scale-eating cichlid (*Perissodus microlepis* Boulenger, 1898) on goldfish scales (Carassius auratus Linnaeus, 1758) (Takeuchi et al., 2012)." (Lines 266-268) add something like "Animal care procedures were approved by the Institutional Animal Care Committee of Nagoya University (permit number #0014) (Takeuchi et al., 2012)." or perhaps "animal care and ethical considerations were made when this video data was collected (Takeuchi et al., 2012)." Even though this information is available in Takeuchi et al., 2012 I think it is worth mentioning in this manuscript.

Reproducibility

The methods are very well explained and aid reproducibility, I only have a couple of minor suggestions for improvements:

In order for this study to function as a stand-alone reproducible work there should be a figure showing the difference between 'righty' and 'lefty' individuals, either photographs or diagrams. This would be a useful addition to figure 3.

Lines 337-338: Is the midline used to determine the angle of approach based on the central coordinate landmark or the absolute length of the goldfish? I imagine these two positions will be (almost?) identical but I think it's worth stating explicitly in the text.

Completeness

The conclusions are supported by the data, there are very clear links between the data, methods, results and conclusions. I do not think there are any flaws in the experimental design that would invalidate any of the results.

As the authors note, it would be interesting to increase the number of experiments per individual to work out whether individuals vary strategy, but I recognise that this is outside of the scope of this particular study.

I do think that there could be some improvements made to the conclusions:

Lines 259-262 "The multiple predatory strategies identified here may contribute to the evolutionary processes that maintain this left-right dimorphism. Investigating the diversity of predation strategies within a species therefore has the potential to provide important ecological and evolutionary insights into complex predator-prey interactions." This touches on the potential wider implications but it would be good to expand on them slightly. How would the multiple predatory strategies identified contribute to the evolutionary processes that maintain this left-right dimorphism?

© 2026. Published by The Company of Biologists under the terms of the Creative Commons Attribution License (https://creativecommons.org/licenses/by/4.0/).

Scholarship

I have no concerns regarding the scholarship of the manuscript, the authors engage appropriately with the wider literature. I found the discussion of neural mechanisms particularly interesting.

<u>Reviewer 2</u>

Comments for the author

This study describes attack trajectories of a scale-eating cichlid. These cichlids, Perissodus microlepis, are a well-known system in which individuals have genetically driven right and left rotated mouth morphology that is linked to whether they attack the left or right flank of prey species. Fractions of right and left attackers evolve as prey learn to avoid whichever morph is more prevalent. Consequently, this system is of great interest.

The current manuscript tests the main hypothesis that the initiation position of an attack approach will also show a handedness. And indeed, there is a tendency for attackers to initiate an attack from their morphologically specialized side when analyzed as a whole. But the sample size is small. For lefty individuals, 17/21 initiated from the left flank and for righty individuals 19/30 initiated from the right flank. Taken together this is significantly more than expected by chance, but I calculated the Chi-Square for just the lefties and that difference is not significant. The sample size is thus marginal for the stated conclusion.

The manuscript also provides some more descriptions of interesting differences in attack categories for rear approaching versus side approaching attackers but concludes that the attack mode does not affect the success rate.

Another potential weakness in this manuscript is that the reference frame for analysis is defined in terms of the prey fish. This makes some sense for analyzing the relative polar position of predators and prey, but it could lead to serious distortions for speeds estimated. The manuscript should be edited to show clearly when the results are given in lab frame and when they are given in prey frame and it would be prudent to provide an estimate for how much that choice of reference frame matters in this case.

Specific comments:

Notes:

Line 80. It would be useful to explain the morphology more precisely. Perhaps "lefty individuals, with mouths twisted clockwise as seen from above, preferentially attack the prey's left flank, while righty, with mouths twisted counterclockwise as seen from above, ones target the right." Or perhaps show an enlarged photo of the two twists and lefty/righty naming convention.

Line 103. More precision in describing "corresponding" would be helpful. i.e. be specific about whether "corresponding" means that lefty fish initiate an attack from the left rear quadrant of the prey.

Line 119-122. 19 out of 30 lefty individuals approached from the left side. Analyzing those by themselves using Chi-square yield a p=0.14. So, analyzing the lefties and righties separately yields the surprising result that perhaps righties attack preferentially but perhaps lefties do not. This highlights a problem with the small sample size in this study.

Line 145-148. I am unsure whether the maximum center of mass speeds referenced here are in prey fish frame or in lab frame. If they are in prey fish frame I worry that the movement of the goldfish makes these speeds difficult to interpret. In any case, it should be clearly stated what reference frame these speeds are given in.

© 2026. Published by The Company of Biologists under the terms of the Creative Commons Attribution License (https://creativecommons.org/licenses/by/4.0/).

Line 296 - 239. In the definition of the coordinate system with respect to the prey, was the coordinate system really redefined at each time point as implied by equations 1 and 2? Or was the prey coordinate system defined at some time at the beginning of the sequence and all transformations were made relative to that initial coordinate system? If the prey coordinate system were redefined at each time point, potentially very large apparent rotations and apparent accelerations of objects could be generated. A reference frame that was shifting at each time point would be non-inertial and would complicate the calculation and interpretation of speeds. Authors should clarify and justify the rotation angle definition. One alternative would be to change the reference frame relative to some t_0.

Line 333-335. Given the possibly temporally changing coordinate system, formulas should be given for the calculations for: the maximum angular velocity, maximum center-of-mass speed, and maximum body flexion amplitude (defined as the maximum deviation angle from a fully extended tail posture) were extracted.

Figure 2. In the top 6 panels in Fig. 2 A, it looks like the coordinate system is apparently being reset at each timepoint. Although the goldfish appears to move very little. Is that correct? Do the fish hardly move? If so, the non-inertial reference frames might not matter very much. In any case, the axes and figure labelling should make clear whether the speeds given are in the rotating and translating fish frame or whether they are expressed in the lab frame.

Fig. 5 What do the boxes indicate? Quartiles? And the lines - ranges?

Reviewer's Responses to Questions

Experimental quality
Are experiments performed using appropriate methods that will answer the question (or test the hypothesis or support the observations) posed by the authors? Is the right tool used for the job?
If 'No', please indicate reasons in Comments for Author box below.
Reviewer #1:
- Yes

Reviewer #2:
- Yes

Were the data analyzed using appropriate statistical tests?
If 'No', please indicate reasons in Comments for Author box below.
Reviewer #1:
- Yes

Reviewer #2:
- Yes

Reproducibility
Were experiments performed using adequate number of biological replicates?
If 'No', please indicate reasons in Comments for Author box below.
Reviewer #1:
- Yes

Reviewer #2:
- Yes

Is there sufficient raw data to assess the rigor of the analysis?
If 'No', please indicate reasons in Comments for Author box below.
Reviewer #1:

© 2026. Published by The Company of Biologists under the terms of the Creative Commons Attribution License (https://creativecommons.org/licenses/by/4.0/).

- Yes

Reviewer #2:
- Yes

Does the methods section provide sufficient detail to permit reproducibility?
If 'No', please indicate reasons in Comments for Author box below.
Reviewer #1:
- Yes

Reviewer #2:
- Yes

Completeness
Are the manuscript's conclusions supported by the data?
If 'No', please indicate reasons in Comments for Author box below.
Reviewer #1:
- Yes

Reviewer #2:
- Yes

Scholarship
Do the authors cite and discuss the merits of data that would argue for and against their conclusion?
If 'No', please indicate reasons in Comments for Author box below.
Reviewer #1:
- Yes

Reviewer #2:
- Yes

Does the manuscript title & abstract accurately reflect the contents of the manuscript, without hyperbole?
If 'No', please indicate reasons in Comments for Author box below.
Reviewer #1:
- Yes

Reviewer #2:
- Yes

First revision

Author response to reviewers’ comments

Reviewer #1:

We are grateful to Reviewer #1 for their valuable comments, which have helped us improve the manuscript considerably. We have considered all the comments in the revised manuscript.

Comment #1

© 2026. Published by The Company of Biologists under the terms of the Creative Commons Attribution License (https://creativecommons.org/licenses/by/4.0/).

After the sentence "We reanalyzed previously recorded high-speed video data of predation behavior of a scale-eating cichlid (*Perissodus microlepis* Boulenger, 1898) on goldfish scales (Carassius auratus Linnaeus, 1758) (Takeuchi et al., 2012)." (Lines 266-268) add something like "Animal care procedures were approved by the Institutional Animal Care Committee of Nagoya University (permit number #0014) (Takeuchi et al., 2012)." or perhaps "animal care and ethical considerations were made when this video data was collected (Takeuchi et al., 2012)." Even though this information is available in Takeuchi et al., 2012 I think it is worth mentioning in this manuscript.

• We revised this as suggested (p. 11, line 278–280).

Comment #2

In order for this study to function as a stand-alone reproducible work there should be a figure showing the difference between 'righty' and 'lefty' individuals, either photographs or diagrams. This would be a useful addition to figure 3.

• We have added photos and description of righty and lefty individual to Fig. 1, which was originally Fig. 3 in the previous manuscript, in the revised version.

Comment #3

Lines 337-338: Is the midline used to determine the angle of approach based on the central coordinate landmark or the absolute length of the goldfish? I imagine these two positions will be (almost?) identical but I think it's worth stating explicitly in the text.

• The midline of the goldfish was defined based on the specified landmarks (snout and midpoint). An explanation of this has been added (p. 14, line 362).

Comment #5

Lines 259-262 "The multiple predatory strategies identified here may contribute to the evolutionary processes that maintain this left-right dimorphism. Investigating the diversity of predation strategies within a species therefore has the potential to provide important ecological and evolutionary insights into complex predator-prey interactions." This touches on the potential wider implications but it would be good to expand on them slightly. How would the multiple predatory strategies identified contribute to the evolutionary processes that maintain this left-right dimorphism?

• Thank you for your comment. The rear and side approaches identified here differed not only in predator positioning, but also in strike timing, locomotor dynamics, and bite location. From the prey’s perspective, these strategies may therefore represent distinct forms of attack that require different escape responses. Such behavioral diversity could increase the unpredictability of predatory attacks and reduce the prey’s ability to anticipate or learn effective escape behaviors. Variation in predatory behavior may contribute to maintaining this left–right dimorphism. An explanation has been added to the manuscript accordingly (p. 10, line 265-271).

Reviewer #2:

We are grateful to Reviewer #2 for the valuable comments and have considered all the comments in the revised manuscript.

Comment #1

The current manuscript tests the main hypothesis that the initiation position of an attack approach will also show a handedness. And indeed, there is a tendency for attackers to initiate an attack from their morphologically specialized side when analyzed as a whole. But the sample size is small. For lefty individuals, 17/21 initiated from the left flank and for righty individuals 19/30 initiated from the right flank. Taken together this is significantly more than expected by chance, but I

© 2026. Published by The Company of Biologists under the terms of the Creative Commons Attribution License (https://creativecommons.org/licenses/by/4.0/).

calculated the Chi-Square for just the lefties and that difference is not significant. The sample size is thus marginal for the stated conclusion.

• We thank the reviewer for this important comment. We also acknowledge that our original statement, that lefty individuals predominantly initiated their approach from the prey's left side (19/30), whereas righty individuals initiated their approach from the right side (17/21), was stronger than what was directly supported by our chi-square test that was applied to the whole data. However, as the reviewer also noted, the chi-square test performed on the entire dataset did reveal a significant association between mouth morphology and the side from which the attack approach was initiated. Further, we reanalyzed the data using a generalized linear mixed model (GLMM). The results of this analysis remained consistent with our conclusion that approach initiation position is associated with mouth asymmetry (coefficient = −1.993, z = −2.964, P = 0.003).Taking the reviewer's concern into account, we revised the text to describe the left-right positioning only as a "tendency" and to state the conclusion supported by the statistical analysis more clearly (p. 6, line 122-126).

Comment #2

Another potential weakness in this manuscript is that the reference frame for analysis is defined in terms of the prey fish. This makes some sense for analyzing the relative polar position of predators and prey, but it could lead to serious distortions for speeds estimated. The manuscript should be edited to show clearly when the results are given in lab frame and when they are given in prey frame and it would be prudent to provide an estimate for how much that choice of reference frame matters in this case.

• We thank the reviewer for this suggestion. All movement variables shown in Figs. 3, 4A, 6C (except for relative angle), and 7B were calculated in the original coordinate space (i.e., lab frame) rather than the prey-aligned coordinate space (i.e., prey frame). Therefore, the coordinate transformation does not introduce the kind of serious distortion raised by the reviewer. To avoid any misunderstanding, we have clarified this point in the Results (p. 6, line 146-148), Methods (p. 13, 322-325), and the legend of Figure 6 (p. 30, line 643-644).

Comment #3

Line 80. It would be useful to explain the morphology more precisely. Perhaps "lefty individuals, with mouths twisted clockwise as seen from above, preferentially attack the prey's left flank, while righty, with mouths twisted counterclockwise as seen from above, ones target the right." Or perhaps show an enlarged photo of the two twists and lefty/righty naming convention.

• We have added a description of the mouth morphology of scale-eating fish (p. 4, line 78-80).

Comment #4

Line 103. More precision in describing "corresponding" would be helpful. i.e. be specific about whether "corresponding" means that lefty fish initiate an attack from the left rear quadrant of the prey.

• We revised this as suggested (p. 5, line 105-106).

Comment #5

Line 119-122. 19 out of 30 lefty individuals approached from the left side. Analyzing those by themselves using Chi-square yield a p=0.14. So, analyzing the lefties and righties separately yields the surprising result that perhaps righties attack preferentially but perhaps lefties do not. This highlights a problem with the small sample size in this study.

• As described in our response to Comment #1 above, we revised the manuscript to describe the observed lateral bias in more cautious terms.

Comment #6

© 2026. Published by The Company of Biologists under the terms of the Creative Commons Attribution License (https://creativecommons.org/licenses/by/4.0/).

Line 145-148. I am unsure whether the maximum center of mass speeds referenced here are in prey fish frame or in lab frame. If they are in prey fish frame I worry that the movement of the goldfish makes these speeds difficult to interpret. In any case, it should be clearly stated what reference frame these speeds are given in.

• The maximum center-of-mass speeds were calculated in the lab frame. We have clarified this point in the revised manuscript by explicitly stating the reference frame used for the speed calculation (p. 6, line 146-148).

Comment #7

Line 296 - 239. In the definition of the coordinate system with respect to the prey, was the coordinate system really redefined at each time point as implied by equations 1 and 2? Or was the prey coordinate system defined at some time at the beginning of the sequence and all transformations were made relative to that initial coordinate system? If the prey coordinate system were redefined at each time point, potentially very large apparent rotations and apparent accelerations of objects could be generated. A reference frame that was shifting at each time point would be non-inertial and would complicate the calculation and interpretation of speeds. Authors should clarify and justify the rotation angle definition. One alternative would be to change the reference frame relative to some t_0.

• We redefined the prey coordinate system independently for each frame. However, these transformed coordinates were used only for the analyses of relative predator-prey position and predation strategy classification. All movement variables were calculated in the original laboratory reference frame. We have clarified this point in the revised manuscript (p.12, lines 308-310, 322–325).

Comment #8

Line 333-335. Given the possibly temporally changing coordinate system, formulas should be given for the calculations for: the maximum angular velocity, maximum center-of-mass speed, and maximum body flexion amplitude (defined as the maximum deviation angle from a fully extended tail posture) were extracted.

・These movement variables were not calculated using the prey-aligned coordinate system. Maximum angular velocity, maximum center-of-mass velocity, and maximum body flexion amplitude were all calculated in the original laboratory coordinate frame (or from body geometry independent of the prey-centered transformation). To avoid confusion, we have clarified this point in the revised manuscript and explicitly stated the reference frame used for these movement variables (p. 6, 146-148).

Comment #9

Figure 2. In the top 6 panels in Fig. 2 A, it looks like the coordinate system is apparently being reset at each timepoint. Although the goldfish appears to move very little. Is that correct? Do the fish hardly move? If so, the non-inertial reference frames might not matter very much. In any case, the axes and figure labelling should make clear whether the speeds given are in the rotating and translating fish frame or whether they are expressed in the lab frame.

・The coordinate system shown in the top panels of Fig. 7A in the revised version was indeed defined independently for each frame, such that the prey's trunk center was positioned at the origin and the trunk center–snout axis was aligned with the positive y-axis. This transformation was used only to visualize and quantify the predator's position relative to the prey. The movement variables reported in this study, including center-of-mass speed and angular velocity, were calculated in the original laboratory reference frame and were therefore not affected by the frame-by-frame coordinate transformation. To avoid confusion, we have clarified in the figure legend and the main text which analyses were performed in the prey-centered frame and which were performed in the laboratory reference frame (p. 6, line 146-148, p. 30, 643-644).

© 2026. Published by The Company of Biologists under the terms of the Creative Commons Attribution License (https://creativecommons.org/licenses/by/4.0/).

Comment #10

Fig. 5 What do the boxes indicate? Quartiles? And the lines - ranges?

・For each boxplot, the bottom and top of the box indicate the 25th and 75th percentiles, respectively; the thick black line indicates the median; and the whiskers extend to the most extreme data points that are not outliers. Outliers are defined as values more than 1.5 times the interquartile range beyond the lower or upper quartile. We have revised the Fig. 3 legend to explain the meaning of the boxes, median lines, whiskers, and outliers (p. 24, line 593-597).

Second decision letter

MS ID#: bio.062699R1

MS Title: Prey capture strategies relate to prey position in the scale-eating cichlid Perissodus microlepis

Authors: Kai Koike; Matasaburo Fukutomi; Yuichi Takeuchi

I am happy to tell you that your manuscript has been accepted for publication in Biology Open, pending our standard publication integrity checks. It was accepted on 15th June 2026.

Reviewer 1

Comments for the author

The revisions have improved the manuscript, which was already very good.

I have no concerns regarding experimental quality, reproducibility, completeness or scholarship, and have no further suggestions or comments.

Reviewer 2

Comments for the author

The authors have made important clarifications in the manuscript, most importantly with respect to the frames of reference used and in the description of mouth morphology. These changes improve the clarity and precision of the manuscript.

Reviewer's Responses to Questions

Experimental quality
Are experiments performed using appropriate methods that will answer the question (or test the hypothesis or support the observations) posed by the authors? Is the right tool used for the job?
If 'No', please indicate reasons in Comments for Author box below.
Reviewer #1:
- Yes

Reviewer #2:
- Yes

Were the data analyzed using appropriate statistical tests?
If 'No', please indicate reasons in Comments for Author box below.

© 2026. Published by The Company of Biologists under the terms of the Creative Commons Attribution License (https://creativecommons.org/licenses/by/4.0/).

Reviewer #1:

- Yes

Reviewer #2:

- Yes

Reproducibility
Were experiments performed using adequate number of biological replicates?
If 'No', please indicate reasons in Comments for Author box below.
Reviewer #1:

- Yes

Reviewer #2:

- Yes

Is there sufficient raw data to assess the rigor of the analysis?
If 'No', please indicate reasons in Comments for Author box below.
Reviewer #1:

- Yes

Reviewer #2:

- Yes

Does the methods section provide sufficient detail to permit reproducibility?
If 'No', please indicate reasons in Comments for Author box below.
Reviewer #1:

- Yes

Reviewer #2:

- Yes

Completeness
Are the manuscript's conclusions supported by the data?
If 'No', please indicate reasons in Comments for Author box below.
Reviewer #1:

- Yes

Reviewer #2:

- Yes

Scholarship
Do the authors cite and discuss the merits of data that would argue for and against their conclusion?
If 'No', please indicate reasons in Comments for Author box below.
Reviewer #1:

- Yes

Reviewer #2:

- Yes

Does the manuscript title & abstract accurately reflect the contents of the manuscript, without hyperbole?
If 'No', please indicate reasons in Comments for Author box below.

© 2026. Published by The Company of Biologists under the terms of the Creative Commons Attribution License (https://creativecommons.org/licenses/by/4.0/).

Reviewer #1:

- Yes

Reviewer #2:

- Yes

© 2026. Published by The Company of Biologists under the terms of the Creative Commons Attribution License (https://creativecommons.org/licenses/by/4.0/).
